# Silicon photonic crystal thermal emitter at near-infrared wavelengths

**DOI:** 10.1038/srep13415

**Published:** 2015-08-21

**Authors:** Bryan J. O’Regan, Yue Wang, Thomas F. Krauss

**Affiliations:** 1 School of Physics & Astronomy, University of St Andrews, St Andrews, KY16 9SS, UK; 2 Department of Physics, University of York, York, YO10 5DD, UK

## Abstract

Controlling thermal emission with resonant photonic nanostructures has recently attracted much attention. Most of the work has concentrated on the mid-infrared wavelength range and/or was based on metallic nanostructures. Here, we demonstrate the experimental operation of a resonant thermal emitter operating in the near-infrared (≈1.5 μm) wavelength range. The emitter is based on a doped silicon photonic crystal consisting of a two dimensional square array of holes and using silicon-on-insulator technology with a device-layer thickness of 220 nm. The device is resistively heated by passing current through the photonic crystal membrane. At a temperature of ≈1100 K, we observe relatively sharp emission peaks with a Q factor around 18. A support structure system is implemented in order to achieve a large area suspended photonic crystal thermal emitter and electrical injection. The device demonstrates that weak absorption together with photonic resonances can be used as a wavelength-selection mechanism for thermal emitters, both for the enhancement and the suppression of emission.

Controlling spontaneous emission is a topic that has fascinated generations of researchers. The topic received a major boost with the introduction of photonic crystals (PhCs), which enabled both the enhancement^1^ and the suppression^2^ of interband emission, e.g. from III-V materials, by orders of magnitude. The next challenge is to extend spontaneous emission control from these relatively narrowband emitters to the ultimate broadband source, i.e. a thermal emitter. Initial work in this area has focused on metallic structures^3^,^4^,^5^,^6^, but many of these tend to be unstable at elevated temperatures with the exception of refractory metals such as tantalum or tungsten that can reach stable temperatures greater than 1000 K^7^,^8^. Furthermore, any resonances in a metallic structure have a low Q factor due to the high broadband absorption of the metal. For example, reference [3] reports a metallic gold structure with an emission peak at 3.85 μm with a Q factor of approximately 10; reference [7] illustrates a three dimensional (3D) metallic PhC with a peak emission at 1.5 μm but with a Q factor ≈1.7. Clearly, these examples highlight the utility of photonic resonances for thermal emission control, but for many applications, a narrower emission line is desirable. Applications for controlled thermal emitters are wide-ranging and include light sources for mid-infrared gas sensing^9^, solar thermophotovoltaics^10^ and broadband emitters for passive radiative cooling^11^. In order to achieve a narrower emission line, one can restrict the thermal emission by using materials with narrow absorption lines, such as intersubband transitions in III-V materials^12^. However, these tend to be available only at limited wavelengths and the materials are not necessarily stable at high temperatures. Here, we propose and demonstrate the use of highly doped silicon as the thermal emission material. Doped silicon exhibits low and controllable optical loss, hence supports higher Q resonances, yet also higher heat stability than standard metals and other dielectrics, especially when coated with suitable thin films such as Al_2_O_3_ or HfO_2_^13^,^14^. Since Kirchhoff’s law states that, in a thermal equilibrium state, the emissivity (ε) is directly related to the absorptivity (α), ε(λ) = α(λ)^15^, the idea we put forward here is to create narrowband thermal emitters by enhancing the absorption resonantly at the desired wavelength, while suppressing it elsewhere, thereby exercising broadband control over the thermal emission spectrum. We show that by introducing a PhC structure into the doped silicon layer, enhanced resonant absorption occurs at the photonic modes of the structure, which generates narrow emission peaks when heated.

## Fabrication

**Figure 1:**
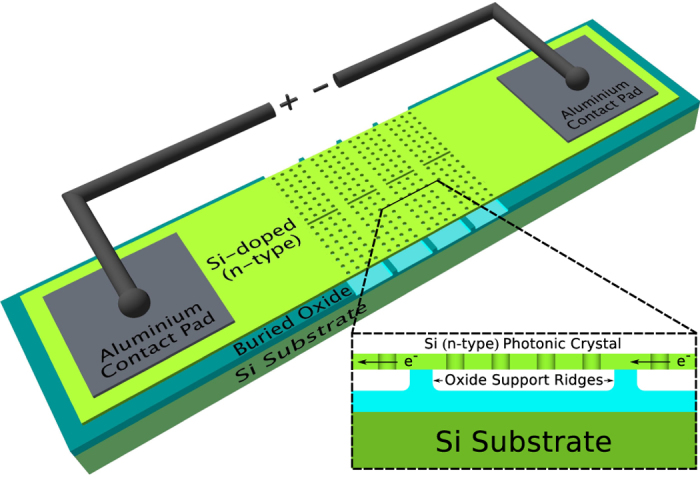
Schematic of the thermal emitter device. The device consists of a n-type doped silicon layer on an SOI wafer, with the PhC structure in the centre and aluminium contact pads for current injection at each side. The inset illustrates a cross sectional view of the device and shows the membraned PhC structure supported on ridges of oxide, which allows for fabrication of large area PhC slabs.

The thermal emitter device consists of a PhC structure etched into the highly doped, 220 nm thick silicon membrane of a silicon-on-insulator (SOI) wafer (SOITEC). Figure 1 shows a schematic of the device, with a cross sectional view of the PhC illustrated in the inset. We apply an electrical bias across the PhC via external aluminium contact pads to resistively heat the membrane. The first step in fabricating the device is to dope the 220 nm thick silicon device layer, which sits on top of a 2 μm layer of buried oxide. As a starting point, we chose a relatively high doping concentration in the 10^20^ cm^−3^ regime. We used solid source diffusion method (Phosphorus Grade PH-950, Saint-Gobain Ceramics, USA) in a furnace with a nitrogen environment at 1000 °C for 45 mins. According to reference [16] for a n-type doping concentration of ≈2.5 × 10^20^ cm^−3^, it results in an absorption coefficient of 4700 cm^−1^ and a refractive index of 3.31 around 1.4 μm.

**Figure 2:**
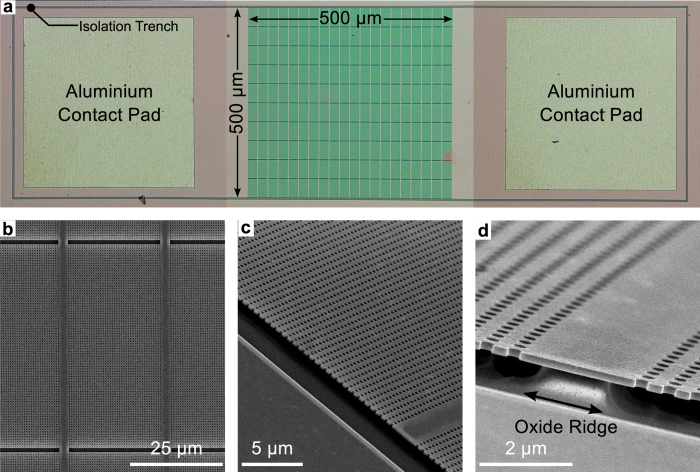
Optical and electron micrograph images of the fabricated thermal emitter device. (**a**) Top-down view of the fabricated device. The image shows the deposited aluminium contact pads on both sides of the PhC structure along with the isolation trench around the entire device. (**b**) SEM image of the PhC structure, showing missing rows of holes in the vertical direction for the support ridges and the gaps in the horizontal direction to allow for thermal expansion of the crystal area. (**c**) SEM image showing the PhC slab suspended above the substrate. (**d**) Zoomed-in SEM image showing the oxide support ridge beneath the PhC slab.

The PhC structure consists of a two dimensional (2D) square array of holes, which was generated by electron beam lithography using the positive e-beam resist AR-P 6200.09 (ALLRESIST, Germany) at a thickness of 350 nm. The pattern was transferred into the silicon layer by dry etching using a reactive ion etcher with a gas ratio of SF_6_:CHF_3_ = 1.00:1.16. Isolation trenches around the device and around the aluminium contact pads were added to provide better electrical isolation. Aluminium contacts were deposited using thermal evaporation. Finally, to improve thermal isolation and to enable high temperature operation, the buried oxide layer beneath the silicon PhC layer was removed using a mixture of hydrofluoric (HF) acid and ammonium fluoride (NH_4_F) (HF:NH_4_F = 1.0:4.8) in order to create a free-standing silicon membrane. Figure 2(a) shows an optical micrograph of the fabricated device. The image shows the deposited aluminium contact pads at both sides of the PhC structure along with the isolation trench around the entire device.

The size of this PhC membrane is a key concern for the operation of the device. If the area is too large, e.g. 100 μm × 100 μm, the membrane collapses after removing the buried oxide layer, thus making contact to the silicon substrate which results in heat leakage. To overcome this limitation, we investigated using support structures consisting of ridges of unetched SiO_2_, and different span lengths between these ridges. We found that support ridges with a width of 2 to 3 μm (four missing holes) could be reproduced reliably during the HF undercut step, and the mechanically stable span length that avoided bowing, even when heated, was approximately 25 μm. We also included 2 μm gaps between different PhC sections to facilitate thermal expansion. Using such a support structure, we were able to fabricate a large area thermal emitter (500 μm × 500 μm), as shown in Fig. 2(a). Three scanning electron micrograph (SEM) images of the fabricated PhC structure, Fig. 2(b–d), highlight the oxide support structure. In Fig. 2(b), the rows of missing holes can be seen in the vertical direction along with thermal expansion gaps in the horizontal direction, which appear dark. Figure 2(c) shows the edge of the PhC structure suspended above the substrate and Fig. 2(d) shows a zoomed-in image of one of the oxide ridges beneath the silicon PhC layer.

### Optical analysis of the PhC structure

**Figure 3:**
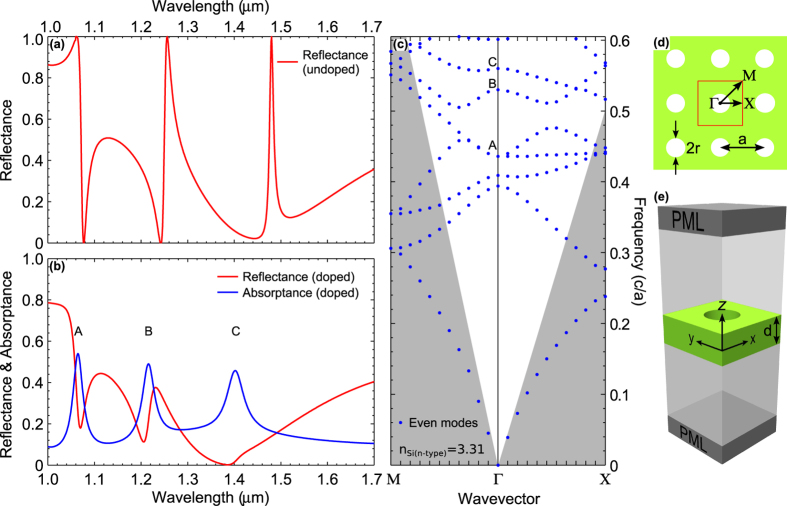
Simulated optical properties of the 2D PhC slab. (**a**) Reflectance spectrum for intrinsic (undoped) silicon at normal incidence obtained by FE simulation. (**b**) Simulated reflectance and absorptance spectrum for a doped (doping concentration of 2.5 × 10^20^ cm^−3^) PhC slab at normal incidence with dispersion as in reference [16] using the FE method. (**c**) Bandstructure computed using 3D FDTD MEEP software for the highly doped PhC structure. The shaded region indicates the area below the light line. (**d**) Illustrates the high symmetry points of the square hole array lattice. (**e**) Illustrates the computational domain used. The parameter values used were a = 605 nm, r = 133 nm and d = 220 nm. The computation domain was the same in both methods except for the source. For FE method the source was a plane wave excited from the top of the domain; while in the FDTD calculation a point source within the slab itself was used.

The optical properties of the photonic structure were first assessed using a 3D finite element (FE) model implemented using the COMSOL^®^ Multiphysics software package^17^. We simulated a normal reflection spectrum for the PhC slab suspended in air both for intrinsic (undoped, n = 3.5) silicon and for the n-type doped (2.5 × 10^20^ cm^−3^) case with dispersion as described in reference [16]. A schematic of the computational domain, with a single unit cell of the PhC slab, is shown in Fig. 3(e). The boundary conditions were set as follows: perfectly matched absorbing layers were used on the top and bottom surfaces with periodic boundary conditions imposed in both the x and y directions perpendicular to the slab. An incident plane wave was excited from the top surface of the computational domain. Figure 3(a) (undoped) and Fig. 3(b) (doped) shows the reflection spectrum for a PhC with period (a) of 605 nm, hole radius (r) of 133 nm and slab thickness (d) of 220 nm. The undoped reflection spectrum, (Fig. 3(a)), shows sharp resonance features. These features represent the quasi-guided resonances of the slab^18^. When doping is introduced, (Fig. 3(b)), the resonances blue-shift due to the reduced refractive index, and they broaden due to the presence of loss. To further assess the PhC structure, 3D finite difference time domain (FDTD) calculations were carried out to construct the photonic bandstructure for the doped case (n = 3.31), using the 3D FDTD software known as MEEP^19^. For the FDTD calculations, the computational domain and boundary conditions were identical to the ones used for the FE method calculations except for the light source. For the FDTD calculations, a dipole source was placed at various positions across the z = 0 plane of the slab. This ensured that the source coupled to all of the possible modes at each k-point and a complete band diagram was obtained, see Fig. 3(c). We observe three optical resonances in reflection for the undoped case, while in the doped case, three absorption resonances. These peaks correspond to points A, B, and C along the Gamma point of the bandstructure. The absorptance of each of the three peaks is approximately 0.5, which is expected from coupled mode theory for such structures when the critical coupling condition applies^20^, in other words, when the coupling loss and the absorption loss in the structure are equal.

## Experimental Results and Discussion

We characterised the device with an optical spectrum analyser (Ando AQ6317B). The emission was imaged onto the facet of the collecting fibre (core diameter of 105 μm, NA = 0.22). With a one-to-one imaging ratio, this NA corresponds to a light collection cone from the sample of 12° around normal. We then applied a bias of up to 78 V, drawing a maximum current of 242 mA.

**Figure 4:**
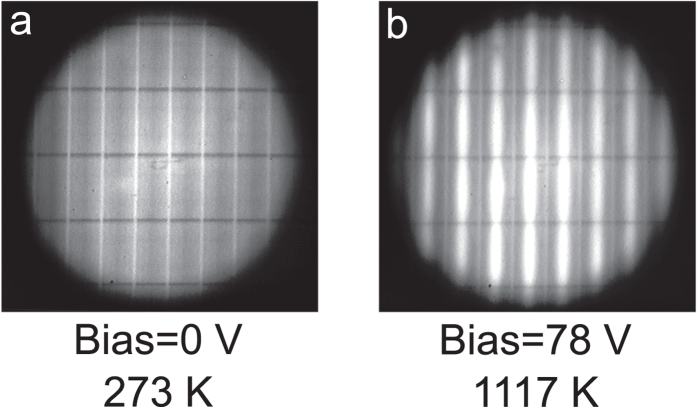
High magnification infrared camera images of the device using a 50x objective. (**a**) No bias applied at room temperature and (**b**) with a bias of 78 V applied reaching a temperature of 1117 K.

Figure 4 shows two zoomed-in infrared camera images of the device imaged with a 50x objective. Figure 4(a) is taken at room temperature while Fig. 4(b) is taken at an elevated temperature of approximately 1117 K with a bias of 78 V applied. The support strips are clearly apparent, as they are slightly cooler and hence darker. The device was kept at elevated temperatures for approximately one hour to complete all the thermal emission spectrum measurements. Afterwards, the photonic crystal was inspected with an optical microscope and no surface defects or any other degradation of the device was visible, except for a small colour change at the very centre of the photonic crystal strips where the temperature reaches its highest point. When the same device was reheated, there was no change in the emission properties suggesting the device is stable at these high temperatures.

Determining the surface temperature of the device is non-trivial, as conventional spectral measurements cannot be used, nor can the fragile membrane be touched by a thermocouple, which would cause a short-circuit and also provide a cooling effect. Even the substrate temperature cannot be used, as it is not directly heated and it has very different thermal emission properties compared to the patterned PhC membrane where the radiation is restricted to a few resonant modes meaning the radiative cooling is suppressed^12^. We therefore exploited the thermo-optic effect and tracked the spectral position of selected PhC resonances in order to determine the actual temperature of the membrane. More details are described in the Methods section. Having established the operating temperature, we can then proceed to spectral measurements.

**Figure 5:**
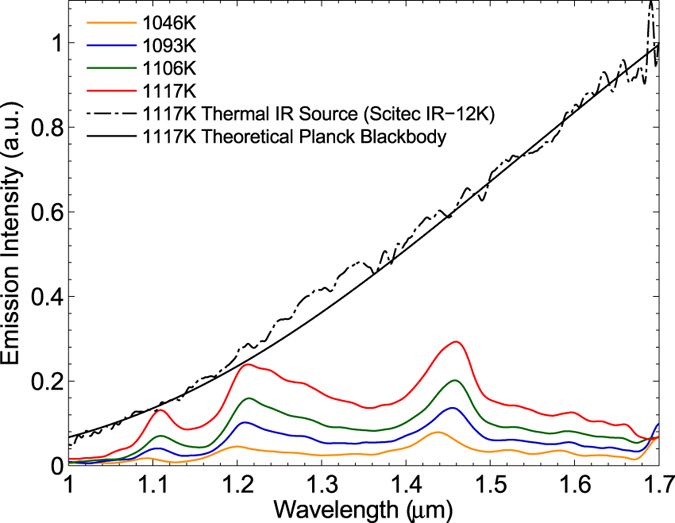
Measured thermal emission spectra. Emission spectra measured with a bias of 72 V, 74 V, 76 V and 78 V, along with the measured blackbody reference spectrum. The blackbody reference curve was obtained from an infrared thermal source (Scitec IR-12K) heated to 1117 K. The light emission was collected over an angle of ≈12° around the normal for both devices. The theoretical Planck blackbody emission at 1117 K is also included.

**Figure 6:**
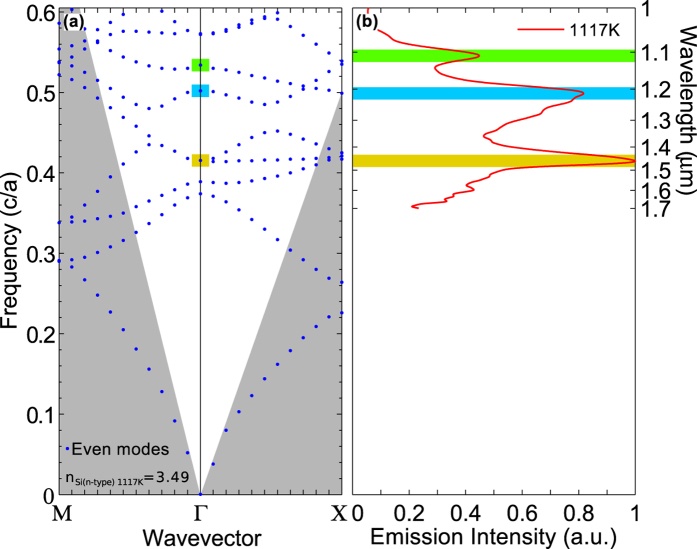
Comparison of the emission spectrum at 1117 K to the corresponding bandstructure. The bandstructure takes into account the index reduction due to the high doping levels and also the index increase due to the high operating temperature. The spectrum on the right hand side is the same as that shown in Fig. 5 for 1117 K.

Figure 5 shows emission spectra with a bias of 72 V, 74 V, 76 V and 78 V. The estimated temperature of each spectrum is 1046 K, 1093 K, 1106 K, and 1117 K, respectively. For each thermal emission spectrum measurement, the device was kept at that temperature for approximately 15 minutes to complete the data acquisition. The black dashed curve is a measured blackbody radiation curve at ≈1117 K taken for reference from an infrared thermal source, while the solid black line indicates a theoretical Planck^21^ blackbody curve at 1117 K. The emission spectra clearly show three distinct emission peaks and we compare the spectrum at 1117 K to the PhC bandstructure in Fig. 6. This bandstructure represents the highly doped PhC structure at high temperature (1117 K), while in contrast to Fig. 3(a), it takes both index reduction due to doping and index increase due to temperature into account. In fact, the reduction in refractive index due to doping (Δn_doped_ = −0.19, at 1.40 μm) and the refractive index increase due to heating (Δn_thermal_ = +0.18, at 1.46 μm, 1117 K) are of very similar magnitude. These three emission peaks correlate excellently with the three bandstructure resonances indicated in Fig. 6. The Q factor of the peak at 1.46 μm is ≈18, with a FWHM of ≈80 nm.

The blackbody reference curve was obtained with an infrared thermal source (Scitec IR-12K) heated to 1117 K. Comparing this curve with the three emission peaks at, 1.11 μm, 1.21 μm and 1.46 μm at the same temperature of 1117 K, we note that the emissivity is 0.5 at the 1.46 μm resonance as expected, while the emissivity of the peaks at shorter wavelengths appear stronger. We associate this apparent discrepancy with differences in the angular radiation properties of the respective emitters. For example, the reference curve represents a Lambertian emitter, while the shorter-wavelength resonances exhibit Fano lineshapes and the 1.46 μm lineshape is Lorentzian, hence the angular emission properties of each type of emitter will be different. However, our measurement only captures a 12° cone, therefore cannot resolve the differences.

## Conclusion

We demonstrate that by controlling the doping concentration in silicon and resonantly enhancing the absorption, accurate control over the material’s absorption coefficient is achieved, which allows engineering of the thermal emission peaks of photonic structures without adding additional absorption features into the material. Compared to previous work on resonant thermal emitters based on metallic structures, we demonstrate a higher Q factor based on the fact that we use doped silicon, which has an intrinsically lower loss. Future work using lower doping densities and higher Q factor resonances should result in even narrower linewidths. Furthermore, by being able to drive the device to higher temperature than comparable metallic devices based on gold or silver and other active dielectric materials, we are able to access the near-infrared wavelength regime around 1.5 μm. Clearly, for the operating temperatures used here, the emission will be stronger in the 3–4 μm wavelength band, but we believe that it is a remarkable proof-of-concept demonstration at all that thermal emission control can be achieved even at 1.5 μm wavelength, and in silicon. The structural stability of the photonic crystal and the stability of the doped silicon material at elevated temperatures is good for short time periods of at least 1 h. To extend the operation of the device to longer time periods, surface passivation layers such as HfO_2_, as in reference [8], may be required to extend the life of the material and to maintain the structural stability of the crystal. Furthermore, we demonstrate that impressive suppression of emission can be achieved off-resonance, as demonstrated by the low emissivity of our structure at around 1.7 μm. This point is particularly noteworthy, as it must be seen in the context of the blackbody background emission, which rises sharply with increasing wavelength. In short, we have demonstrated a significant control of spontaneous thermal emission via photonic resonances alone.

## Methods

### Temperature estimation of emitter

In order to obtain an accurate estimation of the temperature of the PhC thermal emitter, we exploited the thermo-optic effect of silicon. The sample was placed onto an external heater and the surface temperature of the chip was measured using a thermocouple attached to the surface placed away from the PhC. By placing the sample on a heater of high thermal mass, we can assume that the entire sample, including its surface, is in thermal equilibrium. The maximum surface temperature we were able to reach using such an external heater was 740 K. We measured the reflection spectrum at each step of the heating process and tracked the spectral position of the reflection resonance at 1.17 μm. As the PhC heats up, the refractive index of the silicon increases and so does the effective index of the resonance mode, so the mode shifts to longer wavelengths.

**Figure 7:**
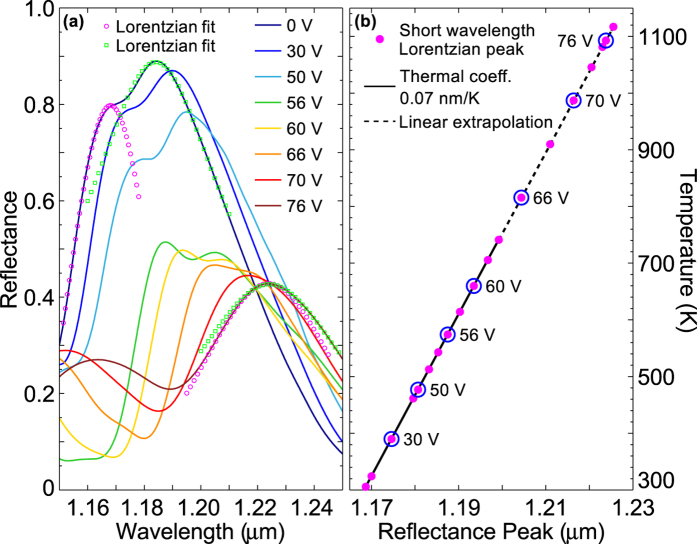
Calibration of the PhC slab temperature by tracking a resonance. (**a**) Measured reflection spectrum for the resistively heated PhC, at room temperature (0 V) and for seven different applied voltages: 30 V, 50 V, 56 V, 60 V, 66 V, 70 V and 76 V. Two Lorentzians are fitted to the reflection resonances (shown here for the case of room temperature and the highest obtained temperature with a voltage of 76 V) in order to track the peak wavelength of the resonances accurately. (**b**) The black solid line represents the thermal coefficient (0.07 nm/K) for the PhC which was calculated using an external heater that reached a maximum surface temperature of 740 K. The linear extrapolation of this coefficient to higher temperatures is also shown. The solid magenta dots represent the peak position of the left hand (short wavelength) reflection resonance (indicated with the magenta Lorentzian fit in panel (a)) as the voltage increases, with the seven reflection resonances from panel (a) highlighted. Using the calculated (and extrapolated) thermal coefficient value, the applied voltage can be accurately mapped onto the corresponding temperature value.

Figure 7(a) shows the measured reflection spectrum for the PhC resistively heated for seven different applied voltages: 30 V, 50 V, 56 V, 60 V, 66 V, 70 V and 76 V. Each reflection curve is a superposition of multiple Lorentzian resonances. Accordingly, we fitted two Lorentzian curves to accurately track the position of each resonant wavelength (shown for the room temperature (0 V) and the high bias 76 V spectra in Fig. 7(a)) for each applied voltage. For the high voltage case of 76 V, the two Lorentzians almost completely overlap and hence have very similar resonant wavelengths. The same fitting and resonance tracking procedure was applied to the reflection data measured with the device on the external heater. This method allows us to determine the relationship between the temperature of the PhC and the resonant reflection wavelength. The black solid line in Fig. 7(b) shows this relationship with a measured thermal coefficient of 0.07 nm/K for the short wavelength resonance, which corresponds to the thermal coefficient of silicon (0.12 nm/K) multiplied by the overlap (Γ ≈ 60%) with the silicon material for this particular mode. Since we were unable to achieve surface temperatures higher than 740 K with the available heater, we extrapolated the line to higher temperatures as a very good linear fit to the data was achieved. Figure 7(b) shows how the temperature for each reflection measurement is determined by using the peak of the reflection resonance for the short wavelength Lorentzian fit mapped onto the thermal coefficient line, with each of the reflection resonances shown in panel (a) individually marked in (b). We estimate an error of +/− 10% for this temperature calibration method.

### Data Availability

All data created during this research are available by request from the University of York Data Catalogue at DOI: 10.15124/af57a781-3768-416b-a5f3-843ef8da1364.

## Additional Information

**How to cite this article**: O’Regan, B. J. *et al.* Silicon photonic crystal thermal emitter at near infrared wavelengths. *Sci. Rep.* **5**, 13415; doi: 10.1038/srep13415 (2015).
